# Enzyme-Specific Redox Stability and Plasticity in Trained Youth Athletes: Longitudinal Analysis of SOD and GPX

**DOI:** 10.3390/ijms27177813

**Published:** 2026-08-31

**Authors:** Jonas Haferanke, Maximilian Dettenhofer, Stefanie Huber, Frauke Mühlbauer, Lisa Baumgartner, Tobias Engl, Thorsten Schulz, Renate Oberhoffer, Sebastian Freilinger

**Affiliations:** Institute of Preventive Pediatrics, Department Health and Sport Sciences, TUM School of Medicine and Health, Technical University of Munich (TUM), 80809 Munich, Germany

**Keywords:** pediatric exercise science, young athletes, antioxidative capacity, superoxide dismutase, glutathione peroxidase, physical activity, training adaptation, oxidative stress, redox plasticity

## Abstract

Resting antioxidant enzyme activity in youth athletes may reflect stable individual redox characteristics and developmentally modulated plasticity. This 12-month longitudinal analysis from the MuCAYA^plus^ study examined the tracking of superoxide dismutase (SOD) and glutathione peroxidase (GPX) in 69 trained athletes aged 10–17 years. Participants underwent repeated assessments of antioxidant enzyme activity, training exposure, body composition, and hematological parameters. Longitudinal tracking was evaluated using Pearson correlation and intraclass correlation coefficients, while change-score regression models tested whether changes in training exposure, growth-related variables, sex interactions, and hematological parameters predicted changes in SOD or GPX. In exploratory analyses, cluster-based redox phenotype stability was assessed using agreement statistics. Over 12 months, SOD decreased whereas GPX increased. GPX showed substantial tracking (r = 0.714, *p* < 0.001; ICC = 0.683), while SOD showed no meaningful tracking (r = 0.057, *p* = 0.644; ICC = 0.053). No statistically supported associations were detected between changes in training-exposure or growth-related measures and enzyme changes. Exploratory erythrocyte-related associations were observed for SOD, whereas leukocyte and platelet changes were unrelated. Cluster-based SOD/GPX phenotype analyses were not robust. These findings are compatible with a comparatively stronger trait-like component for GPX and greater state-like variability for SOD.

## 1. Introduction

Biological adaptation to exercise in youth should not be interpreted as a linear consequence of training exposure alone. During adolescence, physiological systems are shaped by habitual exercise, somatic growth, sex-specific maturation, and substantial changes in body composition [1,2,3]. These developmental processes may alter metabolic demand, oxygen transport, vascular function, and skeletal muscle remodeling, thereby shaping the physiological context in which training adaptation occurs [4]. Redox regulation is particularly relevant in this context because reactive oxygen and nitrogen species (ROS/RNS) are no longer regarded merely as metabolic by-products or mediators of molecular damage [5]. At physiological concentrations, ROS act as pleiotropic signaling molecules that regulate cellular adaptation, mitochondrial remodeling, vascular homeostasis, and stress-response coordination [6,7,8]. Accordingly, resting antioxidant enzyme activity on youth athletes may reflect a dynamic balance between stable individual redox characteristics and short-term response to changing biological and training-related demands.

Superoxide dismutase (SOD) and glutathione peroxidase (GPX) are central enzymatic components of this redox-regulatory network. SOD catalyzes the dismutation of superoxide anions into hydrogen peroxide and oxygen, thereby limiting superoxide-mediated nitric oxide inactivation and downstream oxidative injury [9,10]. GPX reduces hydrogen peroxide and lipid hydroperoxides through glutathione-dependent reactions, contributing to peroxide detoxification, membrane protection and intracellular redox balance [11]. Together, SOD and GPX form a sequential defense axis lining ROS handling to endothelial integrity, skeletal muscle adaptation, and systemic redox homeostasis. Exercise-induced ROS formation can activate redox-sensitive pathways, including Nrf2-dependent antioxidant programs, mitochondrial biogenesis, angiogenic signaling, and skeletal muscle remodeling [12]. However, chronic training does not necessarily produce proportional increases in resting circulating enzyme activity. Repeated exercise exposure may instead establish a regulated redox equilibrium in which enzymatic efficiency, tissue-specific adaptation, or regulatory sensitivity changes without large shifts in the systemic resting level [13,14].

SOD and GPX were interpreted as complementary, but not directly interchangeable, antioxidant markers because they act at different levels of the antioxidant cascade and were assessed in different blood-derived matrices. Plasma SOD reflects a predominantly extracellular measurement, whereas hemolysate-based GPX is more closely related to the erythrocyte-associated antioxidant compartment. Differences in their longitudinal behavior may therefore reflect the biological compartment, assay characteristics, pre-analytical sensitivity, and enzyme regulation rather than intrinsic enzyme properties alone. Erythrocytes are continuously exposed to oxygen flux and hemoglobin autoxidation, generating ROS that are buffered by antioxidant systems including SOD and GPX, catalase, peroxiredoxins, and glutathione-dependent pathways [15,16]. Therefore, hemoglobin, hematocrit, and erythrocyte count may provide developmental context for blood-based redox enzyme activity during adolescence, when erythrocyte mass, blood volume, and oxygen transport capacity are influenced by growth, sex-specific maturation, and training status [17]. Leukocytes and platelets are also redox-active blood cell populations, but their circulating count more likely reflect immune or vascular-homeostasis state variation than stable antioxidant enzyme set points [18,19].

This distinction is especially important in trained children and adolescents. In adults, exercise-related antioxidant responses vary according to enzyme, sex, exercise, intensity, frequency, duration, and baseline redox status [20,21]. In pediatric populations, evidence remains more limited. Recent work has emphasized that redox biology during childhood and adolescence is still insufficiently characterized [22]. Puberty may further modify oxidative and antioxidant profiles through changes in growth velocity, endocrine regulation, insulin sensitivity, body composition, and inflammatory tone [2,23,24]. Thus, chronological age alone is unlikely to capture the relevant biological context. In athletic youth, body compositional factors such as fat-free mass may provide a more informative proxy of developmental redox demand than age or sport discipline alone.

The developmental complexity of youth sport creates a central methodological challenge. Cross-sectional comparisons can identify between-person differences, but they cannot determine whether an athlete’s antioxidant enzyme profile is stable over time or changes in response to individual growth or training fluctuations. This limitation is particularly relevant for resting SOD and GPX. A recent cross-sectional analysis of 203 youth athletes from the MuCAYA^plus^ (Munich Cardiovascular Adaptations in Young Athletes) cohort reported largely stable antioxidant enzyme levels across training volumes, sport disciplines, and age groups, while boys showed higher SOD activity than girls, and cluster analysis identified distinct redox profiles with differing sex and training distributions [25]. These findings suggested that individual and sex-related factors may be more influential than absolute training volume. However, because the design was cross-sectional, it remained unresolved whether the profiles represented stable individual traits, transient physiological states, or both.

A longitudinal framework is therefore required to distinguish trait-like stability from state-like plasticity. Trait stability implies that athletes maintain their relative position within the cohort over time, indicating individual redox set points that persist despite growth and training variation. State-like plasticity implies that athletes deviate from their own set points when training exposure, body composition, or maturation-related characteristics change. These processes are not mutually exclusive; group means may remain stable while individual athletes show meaningful within-person deviations or switch between redox phenotypes. Analytically, this requires models that separate stable between-person differences from time-specific within-person changes because cross-sectional associations cannot be assumed to reflect individual longitudinal adaptation.

In the present study, trait-like and state-like terminology is used operationally to describe relative differences in longitudinal tracking and agreement, rather than to imply formal latent state-trait decomposition. Stronger tracking and agreement were considered compatible with a more trait-like pattern, whereas poor tracking was considered compatible with greater state-like variability.

The present study extends previous cross-sectional work by examining trained youth athletes ages 10–17 years with repeated measurements over 12 months from the first two years of the MuCAYA^plus^ study [26]. The primary aim was to determine whether resting SOD and GPX activities exhibit systematic group-level changes over 12 months in the context of adolescent athletic development, while also assessing whether individual enzyme profiles show trait-like or state-like deviations from personal redox set points. Second, we examined whether within-person changes in training exposure, quantified as weekly training hours and metabolic equivalent of task (MET)-hours per week, and changes in body composition, including fat-free mass and body surface area (BSA), predicted corresponding changes in SOD and GPX. Third, we assessed whether sex modified growth-related associations with antioxidant enzyme changes. In exploratory analyses, we evaluated redox phenotype stability based on the SOD/GPX ratio and examined hematological context variables to determine whether longitudinal redox changes were associated with erythrocyte-related development or immune and vascular-homeostatic state variation. We hypothesized that resting antioxidant enzyme activity would show a measurable trait-like component over 12 months, while allowing for state-like within-person deviations around individual redox set points.

## 2. Results

### 2.1. Study Sample

The longitudinal analytic sample comprised 69 youth athletes with paired measurements at Year 1 and Year 2 for anthropometry, training exposure, antioxidant enzyme activity, and hematological parameters. For body composition variables, paired data were available for 67 participants. Change scores (∆) were calculated as Year 2 minus Year 1 (Table 1). All *p*-values for Year 1 versus Year 2 comparisons reported in Table 1 were obtained using two-sided paired-samples *t*-tests.

Mean age increased from 13.78 ± 1.53 to 14.72 ± 1.54 years, corresponding to a mean change of 0.94 ± 0.13 years. Height increased from 167.71 ± 11.70 to 172.82 ± 11.28 cm, ∆ = 5.11 ± 2.39 cm, and body weight from 54.27 ± 11.91 to 59.67 ± 12.91 kg, ∆ = 5.39 ± 3.32 kg. BMI increased from 19.04 ± 2.33 to 19.73 ± 2.66 kg/m^2^, ∆ = 0.69 ± 0.93 kg/m^2^, and BSA from 1.58 ± 0.22 to 1.68 ± 0.23 m^2^, ∆ = 0.10 ± 0.05 m^2^. Fat-free mass increased from 46.96 ± 11.72 to 52.37 ± 12.13 kg, ∆ = 5.10 ± 3.59 kg.

Weekly training duration increased from 9.96 ± 4.43 to 10.81 ± 3.86 h/week, ∆ = 0.85 ± 3.67 h/week, whilst weekly MET-hours increased from 99.56 ± 48.62 to 111.08 ± 43.93 METh/week, ∆ = 11.52 ± 40.62 METh/week.

SOD activity decreased from 271.24 ± 50.62 to 253.18 ± 45.45 U/mL, ∆ = −18.05 ± 66.09 (95% CI −33.93 to −2.17; *p* = 0.026), corresponding to a small standardized paired effect (d_z_ = −0.27). In contrast, GPX activity increased from 291.11 ± 80.34 to 316.43 ± 82.97 U/L, ∆ = 25.31 ± 61.82 U/L (95% CI 10.46 to 40.16; *p* = 0.001), corresponding to a small-to-moderate standardized paired effect (d_z_ = 0.41). The SOD/GPX ratio decreased from 1.01 ± 0.41 to 0.85 ± 0.29.

Routine hematological parameters remained largely stable over 12 months. Mean differences were minimal for erythrocyte count (∆ − 0.02 ± 0.23/pL), hematocrit (∆0.51 ± 2.67%), hemoglobin (∆0.01 ± 1.47 g/dL) and leukocyte count (∆0.14. ±1.79/nL), whereas platelet count showed a mean decrease of −12.45 ± 34.31 thsd/μL.

### 2.2. Longitudinal Tracking of SOD and GPX

Pearson correlation analyses were used to assess rank-order tracking of antioxidant enzyme activity between Year 1 and Year 2. SOD activity showed no significant correlation between Year 1 and Year 2, r = 0.057, (95% CI −0.182 to 0.290), *p* = 0.644. In contrast, GPX activity showed a significant positive correlation between Year 1 and Year 2, r = 0.714, (95% CI 0.574 to 0.813), *p* < 0.001.

**Figure 1:**
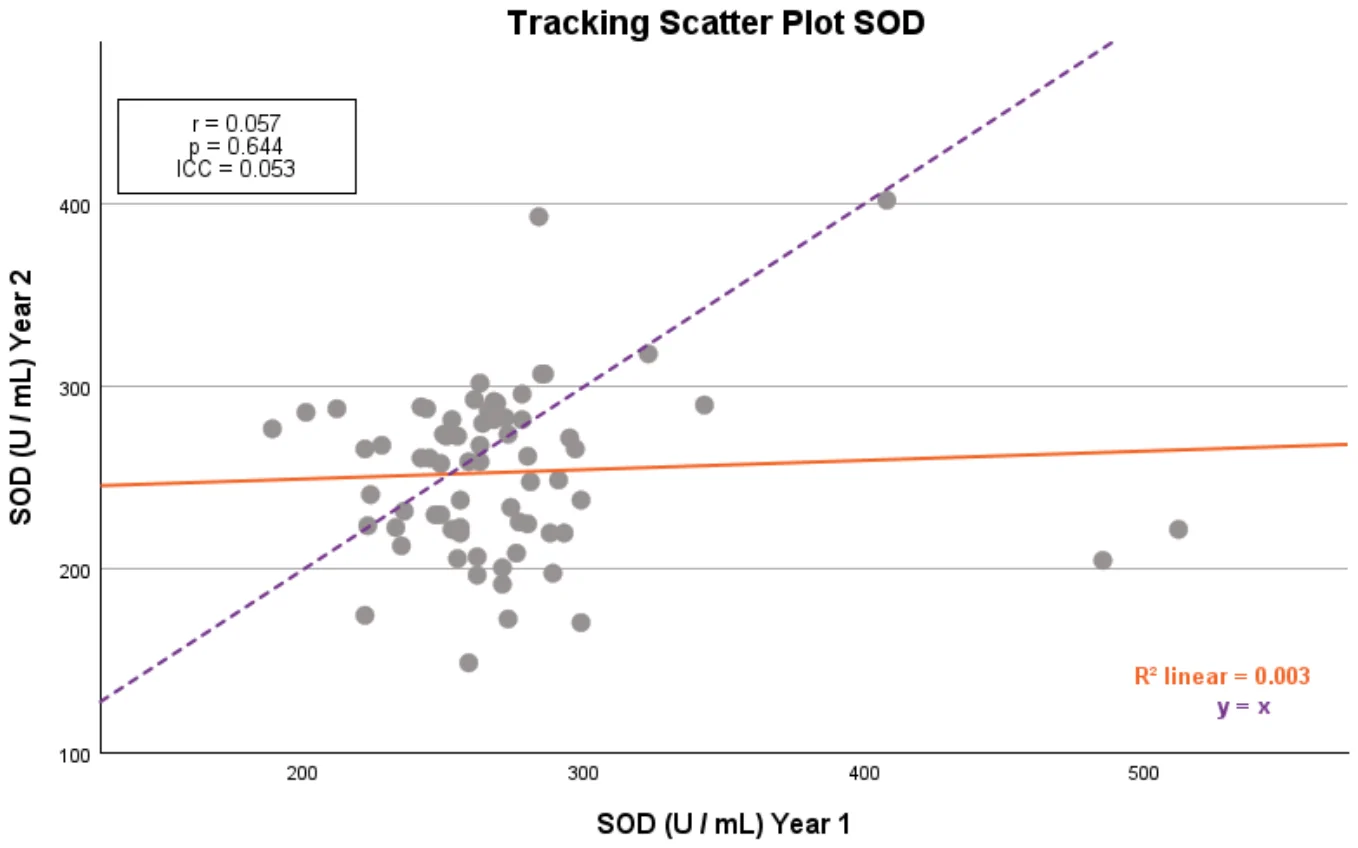
Longitudinal tracking of SOD activity over 12 months.

**Figure 2:**
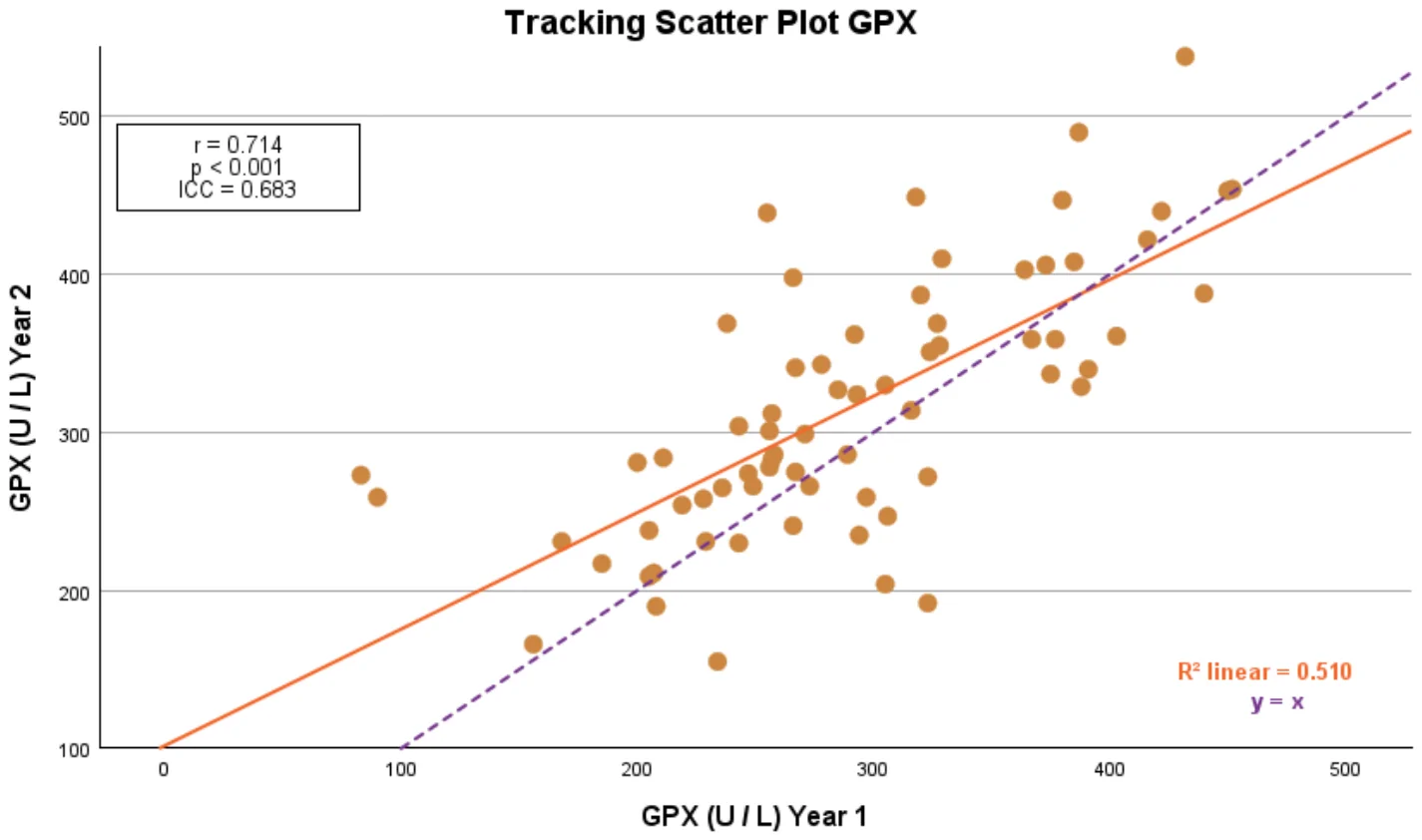
Longitudinal tracking of GPX activity over 12 months.

Intraclass correlation analyses showed poor absolute agreement for SOD, with a single-measure ICC of 0.053 (95% CI −0.169 to 0.275), *p* = 0.322. GPX showed higher absolute agreement, with a single-measure ICC of 0.683 (95% CI 0.501 to 0.801), *p* < 0.001. Tracking scatter plots illustrating the relationship between Year 1 and Year 2 values for SOD and GPX are shown in Figure 1 and Figure 2, respectively.

For the SOD/GPX ratio, Pearson correlation analysis showed moderate tracking between Year 1 and Year 2, r = 0.421, *p* < 0.001. Spearman correlation yielded *ρ* = 0.558, *p* < 0.001. The single-measure ICC for SOD/GPX ratio was 0.368 (95% CI 0.145 to 0.556), *p* < 0.001.

### 2.3. Training Exposure as Predictor of Changes in SOD and GPX

Linear regression models were used to examine whether changes in training exposure predicted changes in antioxidant enzyme activity. For ∆SOD, the model including ∆h/week and ∆METh/week explained 1.8% of the variance and was not statistically significant, R^2^ = 0.018, F = 0.612, *p* = 0.545. Neither ∆h/week nor ∆METh/week was significantly associated with ∆SOD, *p* = 0.425 and *p* = 0.302, respectively.

For ∆GPX, the corresponding model explained 3.8% of the variance and was not statistically significant, R^2^ = 0.038, F = 1.300, *p* = 0.279. Neither ∆h/week nor ∆METh/week was significantly associated with ∆GPX, *p* = 0.706 and *p* = 0.332, respectively.

### 2.4. Growth-Related Predictors and Sex-Specific Moderation of Antioxidant Enzyme Changes

Linear regression models were used to examine whether changes in growth-related and body composition variables predicted longitudinal changes in antioxidant enzyme activity. For ∆SOD, the model including ∆FFM, ∆BSA, and ∆age explained 3.5% of the variance and was not statistically significant, R^2^ = 0.035, *p* = 0.514. None of the individual predictors reached statistical significance: ∆FFM, *p* = 0.938; ∆BSA, *p* = 0.466; ∆age, *p* = 0.249. For ∆GPX, the corresponding model explained 0.5% of the variance without statistical significance, R^2^ = 0.005, *p* = 0.961. None of the included predictors were significantly associated with ∆GPX.

Additional interaction models were used to assess whether sex modified the association between growth-related changes and antioxidant enzyme changes. For ∆SOD, the interaction model explained 2.7% of the variance and was not statistically significant, R^2^ = 0.027, *p* = 0.884. Neither sex × ∆FFM nor sex × ∆BSA was statistically significant, *p* = 0.831 and *p* = 0.726, respectively. For ∆GPX, the corresponding interaction model explained 10.0% of the variance but did not reach overall significance, R^2^ = 0.100, *p* = 0.253. Within this model, sex (*p* = 0.042), ∆BSA (*p* = 0.038), and the sex × ∆BSA interaction (*p* = 0.033) were statistically significant. Because the overall model was not significant, this finding was interpreted as exploratory.

### 2.5. Exploratory Redox Phenotype Analysis Based on the SOD/GPX Ratio

Exploratory cluster analyses based on the SOD/GPX ratio were conducted separately for Year 1 and Year 2. In Year 1, the three-cluster solution yielded the following distribution: Cluster 1, *n* = 2; Cluster 2, *n* = 64; and Cluster 3, *n* = 3. In Year 2, the cluster distribution was more balanced: Cluster 1, *n* = 20; Cluster 2, *n* = 26; Cluster 3, *n* = 23.

Agreement of cluster membership between Year 1 and Year 2 was assessed using Cohen’s kappa. Agreement was low and not statistically significant, κ = 0.030, *p* = 0.469. The association between Year 1 and Year 2 cluster membership was also not significant χ^2^ = 6.84, *p* = 0.166.

### 2.6. Hematological Correlates of Longitudinal Changes in SOD and GPX Activity

Regression models were used to examine whether changes in hematological parameters were associated with longitudinal changes in antioxidant enzyme activity. Erythrocyte-related variables were analyzed separately from leukocytes and platelet counts.

For ∆SOD, the model including ∆erythrocytes, ∆hematocrit, and ∆hemoglobin explained 10.5% of the variance and approached statistical significance, R^2^ = 0.105, F = 2.53, *p* = 0.065. Within this model, ∆erythrocyte count was positively associated with ∆SOD, β = 0.428, *p* = 0.013, while ∆hematocrit was negatively associated with ∆SOD, β = −0.329, *p* = 0.042. ∆Hemoglobin was not significantly associated with ∆SOD, *p* = 0.926. For ∆GPX, erythrocyte-related models explained 5.8% of the variance without statistical significance, R^2^ = 0.058, *p* = 0.270. None of the erythrocyte-related parameters was significantly associated with ∆GPX.

Additional models for ∆leukocyte count and ∆platelet count were used to assess immune-cell and vascular-hemostatic context variables. For ∆SOD, the model explained 4.0% of the variance and was not statistically significant, R^2^ = 0.040, *p* = 0.262. Neither ∆leukocyte count nor ∆platelet was significantly associated with ∆SOD. For ∆GPX, the model explained 0.2% of the variance without statistical significance, R^2^ = 0.002, *p* = 0.927. Neither of the two variables was significantly associated with ∆GPX.

The erythrocyte-related predictors were retained jointly because they represent related but non-equivalent physiological dimensions: cell number, erythrocyte volume fraction, and hemoglobin concentration. Their partial coefficients should nevertheless be interpreted cautiously because shared variance or statistical suppression may affect the direction and precision of individual estimates.

The main statistical findings for longitudinal tracking, predictor models, sex-specific moderation, and hematological correlates are summarized in Table 2.

## 3. Discussion

This longitudinal study investigated whether resting antioxidant enzyme activity in trained youth athletes reflects stable individual redox characteristics or state-like biological variability over 12 months. The principal finding was an enzyme-specific stability pattern. GPX showed substantial longitudinal tracking, whereas SOD showed no meaningful rank-order stability. Thus, GPX appeared to contain a trait-like component of the individual redox phenotype, while SOD was characterized by greater state-like variability. At the group level, SOD decreased and GPX increased, resulting in a lower SOD/GPX ratio at follow-up.

Changes in training exposure, fat-free mass, BSA, and age did not predict changes in SOD or GPX. Sex did not modify SOD-related associations, while the exploratory GPX model suggested a possible sex-specific association with BSA change. Cluster-based phenotype analyses were limited by an imbalanced baseline solution. Hematological analyses indicated an exploratory erythrocyte-related association with SOD changes, whereas leukocyte and platelets were not associated with redox enzyme changes.

### 3.1. Enzyme-Specific Stability: GPX as Trait-like, SOD as State-like

The central finding was the divergent longitudinal stability of SOD and GPX. GPX showed strong 12-month tracking, indicating preservation of inter-individual ranking and supporting a trait-like component of resting GPX activity. In contrast, SOD showed no tracking, suggesting greater within-person variability and a more state-like behavior over the same period. This divergence should be interpreted in light of the distinct enzymatic functions and biological matrices of the two markers. SOD and GPX are not different readouts of the same antioxidant scale, but complementary enzymes operating at different levels of the antioxidant cascade. SOD acts upstream by dismutating superoxide into hydrogen peroxide and was assessed in plasma, whereas GPX acts downstream by reducing hydrogen peroxide and lipid hydroperoxides and was assessed in hemolysates. Thus, the observed difference between SOD and GPX should be interpreted as differential tracking of two matrix- and assay-specific biomarkers, while the proposed biological mechanisms remain explanatory hypotheses rather than direct evidence from the present study. These findings may reflect enzyme-specific differences in redox responsiveness and adaptive regulation. SOD acts upstream in the antioxidant cascade by converting superoxide into H_2_O_2_, thus positioned close to acute ROS generation. Experimental work supports the idea that SOD, particularly MnSOD, can be rapidly redox-responsive, as a single bout of exercise activated SOD gene expression in rat skeletal muscle, with early increases in MnSOD mRNA after treadmill running [27]. Similarly, NOX2-dependent redox signaling after acute exercise has been shown to induce antioxidant and mitochondrial gene transcription, including MnSOD and GPX-related responses [28]. SOD may be particularly sensitive to short-term fluctuations of superoxide production, vascular redox conditions, cellular release, or pre-analytical variability. This could help explain why plasma SOD showed poor 12-month tracking in the present cohort.

GPX, by contrast, acts downstream by reducing H_2_O_2_ and lipid hydroperoxides though glutathione-dependent reactions [11]. Even though acute exercise can also influence GPX-related pathways, blood enzyme responses appear to be enzyme- and time-dependent. For example, after supramaximal anaerobic exercise, antioxidant enzyme activities changed during recovery, and GPX activity was reported to be higher 24 h after exercise [29]. Moreover, because GPX was assessed in hemolysates, it may reflect a more integrated erythrocyte-associated peroxide buffering capacity [30]. The strong tracking of GPX despite a group-level increase may therefore indicate a stable individual set point with superimposed developmental or hematological modulation. Overall, the literature supports a relative, not categorial, distinction; SOD appears more rapidly redox-responsive and state-sensitive, whereas hemolysate-based GPX may capture a more stable, integrated adaptive redox component [6,7].

### 3.2. Training Exposure and Antioxidant Enzyme Changes

Changes in weekly training hours and METh/week did not predict changes in SOD or GPX. This finding suggests that resting antioxidant enzyme changes were not explained by self-reported measures of training exposure, while more granular aspects of training, such as intensity distribution, acute load, recovery status, and periodization, may not have been adequately captured by these variables. It is consistent with contemporary exercise-redox biology, which emphasizes that exercise-induced ROS act as signaling molecules involved in mitochondrial biogenesis, antioxidant gene regulation, angiogenesis and skeletal muscle remodeling, rather than merely reflecting oxidative damage [31,32]. However, these adaptive processes do not necessarily translate into proportional changes in resting circulating SOD or GPX. Recent meta-analytic evidence shows that antioxidant enzymes are heterogenous and depend on exercise intensity, modality, frequency, duration, sex and measurement frequency [14,20]. In adolescent athletes specifically, redox and inflammatory responses to training appear highly variable and are influence by study design, training status, acute versus chronic load and biomarker selection [33]. Thus, in already trained youth, resting SOD and GPX may reflect redox homeostasis and developmental context more than changes in gross weekly training exposure.

### 3.3. Growth, Body Composition, and Sex-Specific Modification

Despite substantial somatic growth, changes in FFM, BSA, and age did not predict changes in SOD or GPX. These variables therefore appear to provide developmental context rather than direct explanatory factors for antioxidant enzyme trajectories. This is plausible because pediatric redox regulation is influenced by puberty, metabolic status, inflammation, and maturation-related physiology, which are not fully captured by chronological age or broad body-size proxies [2,22]. Sex did not modify growth-related associations with SOD. For GPX, an exploratory sex × BSA signal was observed. However, this finding should be interpreted cautiously because the overall interaction model was not significant and the female subgroup was small. One possible biological explanation is that GPX was measured in hemolysates and may therefore be more closely related to erythrocyte-associated peroxide detoxification. During adolescence, BSA change may reflect somatic growth and maturation-related changes in blood volume, erythrocyte development, and oxygen-transport physiology, which may differ between boys and girls. Accordingly, the observed sex × BSA signal should be considered hypothesis-generating and requires confirmation in larger sex-balanced cohorts with direct maturation assessment. Nevertheless, age- and sex-specific dependent development of hemoglobin, blood volume, oxygen transport, and aerobic power supports the biological plausibility of sex-specific redox maturation [17,34].

### 3.4. Redox Phenotype Analysis

Exploratory clustering based on the SOD/GPX ratio did not yield a robust longitudinal phenotype structure. The baseline cluster solution was highly imbalanced, whereas the Year 2 solution was more evenly distributed, and the agreement between time points was poor. This should not be interpreted as definitive evidence of biological phenotype switching. Rather, it indicates that unsupervised clustering of a crude ratio was not sufficiently stable for transition modeling in this sample. This is methodologically relevant because redox biomarkers a matrix-, assay-, and context-dependent, and simple combinations of individual biomarkers may not adequately represent systemic redox balance [13]. Moreover, SOD and GPX are measured on different biological scales and reflect different compartments of the antioxidative cascade. Consequently, the crude SOD/GPX ratio should be interpreted only as an exploratory approximation of relative SOD-to-GPX balance, not as a validated quantitative marker of global antioxidant capacity. Future work should consider standardized redox dominance indices, such as zSOD − zGPX, predefined phenotype thresholds, or externally validated classification approaches.

### 3.5. Hematological Context of Redox Changes

Hematological analyses indicated a selective erythrocyte-related signal for SOD, while erythrocyte-related variables did not independently predict GPX. This is biologically plausible, as erythrocytes represent a major blood redox compartment. Oxygen transport and hemoglobin autoxidation generate superoxide and hydrogen peroxide, which are buffered by the antioxidative cascade [16,35]. Because SOD was measured in plasma, the erythrocyte-related associations with ΔSOD should not be interpreted as direct evidence of erythrocyte SOD activity, but rather as an indication that plasma SOD variability may be coupled to broader oxygen-transport physiology. Conversely, hemolysate-based GPX may be more directly influenced by erythrocyte-associated redox biology, although erythrocyte-related variables did not independently predict ΔGPX in the present models. The associations of ∆erythrocyte count and ∆hematocrit with ∆SOD may therefore reflect coupling between SOD variability and oxygen-transport physiology. However, erythrocyte count, hematocrit, and hemoglobin capture different hematological dimensions and may be affected by plasma volume, therefore their divergent coefficients should be interpreted cautiously. Leukocyte and platelet counts were not associated with SOD or GPX changes. This does not exclude immune or platelet redox biology, as leukocyte ROS generation and platelet ROS signaling are primarily activation-dependent rather than count-dependent [19,36]. Thus, redox enzyme changes were not explained by generalized immune-cell or vascular-hemostatic cell count variation.

#### Limitations

Several limitations should be acknowledged. First, the sample size was modest. Although 69 participants with paired longitudinal data provide valuable information in a pediatric cohort, statistical power was limited, particularly for interaction models, cluster-based phenotype analyses, and hematological subgroup interpretations. The unequal sex distribution, with fewer girls than boys, further limits the robustness of sex-specific analyses and increases the risk that interaction estimates were influenced by individual observations.

Second, the study included only two time points. While a 12-month interval is suitable for assessing annual developmental change, two measurements cannot capture nonlinear trajectories, pubertal tempo, seasonal effects, short-term training adaptations, or transient redox fluctuations. More frequent sampling would be required to distinguish stable individual set-points form temporary biological deviations more precisely.

Third, maturation was assessed indirectly. Age, FFM, BSA, and body composition provide relevant developmental context, but they do not directly measure pubertal stage or endocrine maturation. The absence of Tanner staging, hormonal markers, or other maturational indices limits the interpretation of sex- and growth-related findings, particularly because adolescent redox regulation may be influenced by pubertal processes not captured by anthropometry alone.

Fourth, training exposure was assessed using weekly training hours and METh/week. Although practical and standardized, these measures do not fully capture training intensity distribution, internal load, acute training stress, recovery, competition phase or periodization. Therefore, the absence of training-load associations should not be interpreted as evidence that exercise exposure is irrelevant to redox regulation, but rather that the available training exposure metrics did not explain resting enzyme changes.

Fifth, redox assessment was limited to resting SOD and GPX. These enzymes provide important information on antioxidant activity but do not represent the full redox network. Measures of ROS production, oxidative damage, glutathione status, catalase, peroxiredoxins, Nrf2 signaling, inflammatory cytokines, nitric oxide bioavailability, or post-exercise redox responses were not available.

Sixth, SOD and GPX were assessed in different biological matrices. SOD was measured in plasma, whereas GPX was measured in hemolysates, which may reflect different compartments and pre-analytical sensitivities. Interpretation of GPX would be strengthened by clarification of normalization to hemoglobin or another erythrocyte-related denominator.

Seventh, run-specific intra-assay and inter-assay coefficients of variation, analytical platform identifiers, freeze–thaw history, and cross-year batch information were not available in the investigator-level dataset because analyses were performed within the standardized workflow of an external accredited laboratory. Consequently, the contribution of analytical variability to the observed longitudinal enzyme differences cannot be quantified separately. GPX was reported in U/L of hemolysate.

Eighth, change-score regressions are sensitive to measurement error and regression to the mean. They were retained because the secondary research question concerned associations between concurrent within-person changes, but the results should not be interpreted causally.

Ninth, because multiple secondary and exploratory analyses were performed without formal multiplicity adjustment, isolated statistically significant findings should be interpreted cautiously and require independent confirmation.

Finally, hematological analyses were limited to routine cell counts and concentration markers, which do not capture leukocyte activation, oxidative burst capacity, platelet activation, or platelet-derived ROS. The cluster-based phenotype analysis was also limited by an imbalanced baseline solution. In addition, unmeasured factors such as diet, antioxidant supplementation, selenium and iron status, menstrual cycle phase, and sleep may have influenced redox biology and should be considered in future longitudinal studies.

## 4. Materials and Methods

This longitudinal analysis uses repeated-measures data from the first two study years of the MuCAYA^plus^ study, a prospective single-center cohort study conducted at the Institute of Preventive Pediatrics, Technical University of Munich (TUM). It is designed as a three-year observational cohort study with annual follow-up examinations to investigate cardiovascular and systemic adaptations to physical activity in youth athletes. All study visits, including clinical assessments, anthropometric and body composition measurements, training-related questionnaires, and laboratory analyses were conducted at the study center using standardized procedures. For the present paper, baseline and 12-month follow-up data were analyzed to examine longitudinal changes in resting antioxidant enzyme activity and their association with changes in training exposure and body composition. Data collection for these analyses took place between November 2023 and November 2025. The full study protocol, including detailed information on recruitment, eligibility criteria, examination procedures, and outcome assessment, has been published previously [26]. The MuCAYA^plus^ study was prospectively registered at ClinicalTrials.gov under the identifier NCT06259617.

### 4.1. Participants

Participants were children and adolescents enrolled in the MuCAYA^plus^ study through sports medical examinations at the outpatient clinic of the Institute of Preventive Pediatrics. At baseline, eligible participants were 10–16 years of age, across the first two annual study visits, the range extended to 10–17 years. Inclusion criteria comprised regular participation in a primary sports discipline, club affiliation, active participation in competitive sport and a weekly training volume of at least three hours. To reduce the influence of acute exercise-induced changes, all assessments were scheduled at least 12 h after the last intensive training session. Participation in the MuCAYA^plus^ further required willingness to attend annual follow-up examinations over the three-year study period. Exclusion criteria included acute infections, current injuries, chronic diseases, and missing written informed consent. The longitudinal analytic sample comprised all 69 participants with valid SOD and GPX measurements at both study visits. The number of paired measurements was limited by discontinuation of the specific SOD and GPX analyses by the external laboratory during the study period rather than by additional participant-level exclusion criteria. Paired body-composition data were available for 67 participants.

### 4.2. Ethical Considerations

The study protocol was approved by the Ethics Committee of the Technical University of Munich (protocol code: 2023-426-S-DFG-SB; date of approval: 13 October 2023). Prior to enrollment, participants and their legal guardians received written and verbal study information and written informed consent was obtained from both in accordance with age-appropriate ethical standards.

### 4.3. Blood Sampling and Laboratory Analyses

Fasting venous blood samples were collected in the morning under standardized conditions by trained physicians and analyzed by an external accredited laboratory (SYNLAB MVZ Labor, Munich, Germany). Enzymatic antioxidant activity was determined using standardized, quality controlled, photometric assays. Plasma SOD activity was assessed based on the inhibition of formazan dye formation. Superoxide radicals generated by the xanthine/xanthine oxidase system react with I.N.T. tetrazolium salt, while SOD reduces this reaction by catalyzing superoxide dismutation. GPX activity was measured in hemolysates using a coupled enzymatic assay in which GPX catalyzes the oxidation of reduced glutathione by cumene hydroperoxide. Oxidized glutathione is subsequently reduced by glutathione reductase with concomitant oxidation of NADPH. To preserve GPX activity and avoid interference from other peroxidases, samples were stabilized before addition of Drabkin’s reagent, which inactivated non-GPX peroxidase activity. SOD and GPX were therefore treated as matrix- and assay-specific biomarkers rather than directly comparable measures of total antioxidant capacity. GPX was reported in U/L of hemolysate, and no additional normalization to hemoglobin or another erythrocyte-related denominator was performed.

### 4.4. Assessment of Body Composition

Body weight and body composition were assessed by bioelectrical impedance analysis using the InBody 270 body composition analyzer (InBody Europe, Eschborn, Germany). Height, waist circumference, and hip circumference were measured to the nearest 0.1 cm [37]. Body mass index (BMI) and body surface area (BSA) were calculated [38].

### 4.5. Assessment of Physical Activity

Physical activity and sport-related characteristics were assessed using the validated Motorik-Modul Physical Activity Questionnaire (MoMo-PAQ) [39,40]. The questionnaire captured training history, primary sport discipline, weekly training duration, and competition participation. To enable standardized comparison of activity exposures across sports, weekly metabolic equivalent of task hours (METh/week) were calculated, integrating both activity intensity and duration.

### 4.6. Statistical Analyses

Statistical analyses were conducted using SPSS v30.0.0.0 (IBM Corp., Chicago, IL, USA). Continuous variables are presented as mean ± standard deviation (SD), and categorial variables as absolute and relative frequencies. Longitudinal change scores were calculated as ∆ = Year 2 − Year 1, with positive values indicating increase over the 12-month observation period. Analyses were conducted using available data for each variable, missing values were not imputed.

Group-level changes from Year 1 to Year 2, including the *p*-values reported in Table 1, were assessed using two-sided paired-samples *t*-tests within the total sample and the respective sex-stratified groups. For the principal longitudinal changes in SOD and GPX, 95% confidence intervals for the mean paired differences and Cohen’s (d_z_) effect sizes were additionally calculated. Cohen’s (d_z_) was defined as the mean paired difference divided by the standard deviation of the paired differences.

Longitudinal tracking of SOD and GPX was assessed using Pearson correlation coefficients between Year 1 and Year 2 values. Absolute agreement across time points was additionally evaluated using single-measure intraclass correlation coefficients (ICC) with 95% confidence intervals. For the SOD/GPX ratio, Pearson and Spearman correlations were calculated, and ICCs were used to assess agreement.

To examine whether changes in training exposure predicted antioxidant enzyme changes, linear regression models were fitted with ∆SOD and ∆GPX as dependent variables and ∆training h/week and ∆METh/week as predictors. Additional regression models tested whether growth- and body composition-related changes, including ∆fat-free mass (FFM), ∆body surface area (BSA), and ∆age, predicted ∆SOD and ∆GPX. Sex-specific moderation was examined using interaction models including sex × ∆FFM and sex × ∆BSA terms.

Exploratory redox phenotype analyses were performed using the SOD/GPX ratio. Cluster membership was determined separately for Year 1 and Year 2, and longitudinal agreement was assessed using Cohen’s kappa and chi-square testing. Because of imbalanced cluster distribution, phenotype transition analyses were interpreted descriptively.

The analytical hierarchy comprised group-level changes and longitudinal tracking and agreement of SOD and GPX as principal analyses, training- and growth-related regression models as secondary analyses, and sex-interaction, hematological, and ratio-based phenotype analyses as exploratory. No multiplicity adjustment was applied to the exploratory analyses. Exact *p*-values are reported, and these findings were interpreted as hypothesis generating.

Hematological variables were analyzed as exploratory context parameters. Separate regression models examined associations of ∆SOD and ∆GPX with erythrocyte-related parameters, including ∆erythrocyte count, ∆hematocrit, and ∆hemoglobin, and with state-related blood cell parameters, including ∆leukocyte count and ∆platelet count.

All tests were two-sided, *p* < 0.05 considered as statistically significant. Exploratory analyses were interpreted as hypothesis generating.

## 5. Conclusions

This longitudinal study indicates enzyme-specific stability of resting antioxidant enzyme activity in trained youth athletes. Over 12 months, GPX showed substantial tracking, supporting a trait-like component of the individual redox phenotype, whereas SOD showed poor tracking and greater state-like variability. Accordingly, the trait-like and state-like interpretations should be understood as operational hypotheses derived from relative longitudinal tracking and agreement rather than as formal evidence of latent biological traits or states. Changes in training exposure, body composition, and broad growth proxies did not explain changes in either enzyme. Sex did not modify SOD trajectories, while an exploratory sex-specific association between BSA change and GPX requires confirmation. Cluster-based phenotyping was not sufficiently robust for transition modelling. Hematological analyses suggested that erythrocyte-related changes may contribute to SOD variability, whereas leukocyte and platelet counts were unrelated. Overall, resting redox enzyme profiles in youth athletes are not simple reflections of training volume or sport participation. Larger multi-time point studies with direct maturation assessment and expanded redox, hematological and recovery markers are needed to clarify trait- and state-like redox regulation during adolescent athletic development.

## Figures and Tables

**Table 1:** Study characteristics and antioxidative markers Year 1 vs. Year 2.

Variable	Group	*n*	Year 1 (M ± SD)	Year 2 (M ± SD)	Delta (M ± SD)	*p*
Age (years)	Total	69	13.78 ± 1.53	14.72 ± 1.53	+0.93 ± 0.13	**<0.001**
	Boys	53	13.92 ± 1.55	14.86 ± 1.57	+0.94 ± 0.14	**<0.001**
	Girls	16	13.30 ± 1.36	14.24 ± 1.35	+0.93 ± 0.05	**<0.001**
Body mass (kg)	Total	69	54.27 ± 11.91	59.67 ± 12.91	+5.39 ± 3.32	**<0.001**
	Boys	53	55.06 ± 11.84	60.81 ± 12.74	+5.74 ± 3.35	**<0.001**
	Girls	16	51.66 ± 12.17	55.90 ± 13.15	+4.23 ± 3.05	**<0.001**
Body Height (cm)	Total	69	167.71 ± 11.70	172.82 ± 11.28	+5.11 ± 2.39	**<0.001**
	Boys	53	169.09 ± 12.08	174.63 ± 11.62	+5.54 ± 2.23	**<0.001**
	Girls	16	163.15 ± 9.24	166.80 ± 7.65	+3.65 ± 2.41	**<0.001**
BMI (kg/m^2^)	Total	69	19.04 ± 2.33	19.73 ± 2.66	+0.69 ± 0.93	**<0.001**
	Boys	53	18.99 ± 2.05	19.67 ± 2.18	+0.67 ± 0.91	**<0.001**
	Girls	16	19.19 ± 3.18	19.95 ± 3.94	+0.76 ± 0.99	**0.008**
BSA (m^2^)	Total	69	1.58 ± 0.22	1.68 ± 0.23	+0.10 ± 0.05	**<0.001**
	Boys	53	1.60 ± 0.22	1.71 ± 0.23	+0.10 ± 0.05	**<0.001**
	Girls	16	1.52 ± 0.21	1.60 ± 0.20	+0.07 ± 0.04	**<0.001**
FFM (kg)	Total	67	46.96 ± 11.72	52.37 ± 12.13	+5.10 ± 3.59	**<0.001**
	Boys	52	48.44 ± 12.25	54.57 ± 12.42	+5.88 ± 3.60	**<0.001**
	Girls	15	41.83 ± 8.02	45.08 ± 7.66	+2.40 ± 1.85	**<0.001**
Training duration (h/week)	Total	69	9.96 ± 4.43	10.81 ± 3.86	+0.85 ± 3.67	0.059
	Boys	53	9.53 ± 3.60	10.74 ± 3.76	+1.21 ± 3.66	**0.020**
	Girls	16	11.37 ± 6.44	11.02 ± 4.29	−0.34 ± 3.53	0.700
Training intensity (METh/week)	Total	69	99.56 ± 48.62	111.08 ± 43.93	+11.52 ± 40.62	**0.021**
	Boys	53	94.85 ± 39.77	109.81 ± 41.70	+14.96 ± 40.67	**0.010**
	Girls	16	115.18 ± 69.96	115.27 ± 51.93	+0.09 ± 39.56	0.992
SOD (U/mL)	Total	69	271.24 ± 50.62	253.18 ± 45.45	−18.05 ± 66.09	**0.026**
	Boys	53	278.66 ± 54.08	257.64 ± 47.02	−21.01 ± 70.34	**0.034**
	Girls	16	246.68 ± 25.30	238.43 ± 37.42	−8.25 ± 50.14	0.520
GPX (U/L)	Total	69	291.11 ± 80.34	316.43 ± 82.97	+25.31 ± 61.82	**0.001**
	Boys	53	289.49 ± 87.52	315.60 ± 87.75	+26.11 ± 61.04	**0.003**
	Girls	16	296.50 ± 51.60	319.18 ± 67.12	+22.68 ± 66.34	0.192
SOD/GPX Ratio	Total	69	1.01 ± 0.41	0.85 ± 0.29	−0.16 ± 0.39	**0.001**
	Boys	53	1.06 ± 0.45	0.88 ± 0.31	−0.18 ± 0.42	**0.003**
	Girls	16	0.85 ± 0.16	0.78 ± 0.25	−0.07 ± 0.25	0.302
Erythrocytes (/pL)	Total	69	5.08 ± 0.35	5.06 ± 0.39	−0.02 ± 0.23	0.468
	Boys	53	5.16 ± 0.32	5.17 ± 0.33	0.01 ± 0.18	0.941
	Girls	16	4.82 ± 0.30	4.73 ± 0.41	−0.09 ± 0.34	0.292
Hematocrit (%)	Total	69	42.67 ± 2.89	43.19 ± 3.34	0.51 ± 2.67	0.115
	Boys	53	43.10 ± 2.91	43.82 ± 3.31	0.72 ± 2.51	**0.040**
	Girls	16	41.72 ± 2.40	41.09 ± 2.54	−0.18 ± 3.16	0.822
Hemoglobin (g/dL)	Total	69	14.51 ± 1.00	14.51 ± 1.64	0.01 ± 1.47	0.961
	Boys	53	14.71 ± 1.02	14.73 ± 1.77	0.01 ± 1.61	0.946
	Girls	16	13.84 ± 0.58	13.83 ± 0.83	−0.01 ± 0.87	0.955
Leukocytes	Total	69	5.73 ± 1.23	5.87 ± 1.66	0.14 ± 1.79	0.526
(/nL)	Boys	53	5.64 ± 1.15	5.66 ± 1.24	0.02 ± 1.24	0.886
	Girls	16	6.05 ± 1.46	6.56 ± 2.56	0.51 ± 3.00	0.506
Platelets	Total	69	280.45 ± 60.28	268.00 ± 54.78	−12.45 ± 34.31	**0.004**
(thsd/μL)	Boys	53	274.74 ± 62.06	264.32 ± 58.63	−10.42 ± 35.87	**0.039**
	Girls	16	299.38 ± 51.25	280.19 ± 38.47	−19.19 ± 28.50	**0.017**

BMI, body mass index; BSA, body surface area; FFM, fat-free mass; MET, metabolic equivalent of task; SOD, superoxide dismutase; GPX, glutathione peroxidase. Significant results are marked in bold.

**Table 2:** Summary of longitudinal tracking, predictor models, moderation analyses, and hematological correlates of SOD and GPX changes.

Research Question	SOD	GPX	Main Findings
Longitudinal tracking	r = 0.057, *p* = 0.644; ICC = 0.053	r = 0.714, *p* < 0.001; ICC = 0.683	GPX, but not SOD, showed substantial 12-month tracking
Training exposure predictors	R^2^ = 0.018, *p* = 0.545; Δh/week and ΔMETh/week not significant	R^2^ = 0.038, *p* = 0.279; Δh/week and ΔMETh/week not significant	Training exposure changes did not predict enzyme changes
Growth-related predictors	R^2^ = 0.035, *p* = 0.514; ΔFFM, ΔBSA, Δage not significant	R^2^ = 0.005, *p* = 0.961; predictors not significant	Growth-related changes did not predict enzyme changes
Sex-specific moderation	R^2^ = 0.027, *p* = 0.884; no significant interactions	R^2^ = 0.100, *p* = 0.253; sex × ΔBSA *p* = 0.033	No SOD moderation; exploratory GPX sex × BSA signal
Exploratory SOD/GPX phenotype analysis	/	Y1 clusters: 2/64/3; Y2 clusters: 20/26/23; κ = 0.030, *p* = 0.469; χ^2^ = 6.84, *p* = 0.166	Ratio-based classification was not longitudinally robust
Erythrocyte-related hematology	R^2^ = 0.105, *p* = 0.065; Δerythrocytes β = 0.428, *p* = 0.013; Δhematocrit β = −0.329, *p* = 0.042	R^2^ = 0.058, *p* = 0.270; no predictor significant	Exploratory erythrocyte-related association with SOD only
Leukocytes and platelets	R^2^ = 0.040, *p* = 0.262	R^2^ = 0.002, *p* = 0.927	Leukocyte and platelet changes were unrelated to enzyme changes

## Data Availability

The datasets presented in this article are not readily available due to privacy and ethical restrictions. Requests to access the datasets should be directed to the corresponding authors.

## Abbreviations

The following abbreviations are used in this manuscript:

ROS	reactive oxygen species
RNS	reactive nitrogen species
SOD	superoxide dismutase
GPX	glutathione peroxidase
Nrf2	nuclear factor erythroid 2-related factor 2
MuCAYA^plus^	Munich Cardiovascular Adaptations in Young Athletes Plus
MET	metabolic equivalent of task
TUM	Technical University of Munich
NADPH	nicotinamide adenine dinucleotide phosphate
BMI	body mass index
BSA	body surface area
MoMo-PAQ	Motorik Modul Physical Activity Questionnaire
FFM	fat-free mass
H_2_O_2_	hydrogen peroxide
MnSOD	manganese-superoxide dismutase
NOX2	NADPH oxidase 2
